# A novel technique of a new cannulated screw for treatment of inferior pole patellar fractures: a finite element study

**DOI:** 10.1186/s13018-023-04299-y

**Published:** 2023-10-24

**Authors:** Mingmang Pan, Nuo Yin, Li Du, Feng Xue, Yuchun Shen, Liang Ding

**Affiliations:** Department of Orthopaedics, Shanghai Jiaotong University Affiliated Sixth People Hospital South Campus: Shanghai Fengxian Central Hospital, Shanghai, 201499 China

**Keywords:** Patellar fracture, Inferior pole, Finite element, Ding’s screw

## Abstract

**Objective:**

We invented a new cannulated screw with holes on the tail, which called Ding’s screw. The goal of this study was to evaluate the biomechanical outcomes of this new screw with tension band wiring for the treatment of inferior pole patellar fractures in a finite element model.

**Methods:**

We conducted a finite element biomechanical study using two fixation methods: Ding’s screw and tension band wiring (DSTBW) and cannulated screws and tension band wiring (CSTBW). Two methods were simulated to fix the inferior pole patellar fracture in a finite element model. The relative displacement and stress distribution were analyzed and compared.

**Result:**

There were less displacement and stress distribution of DSTBW in different knee movement (30°, 60°, 90°, 120°) when compared to CSTBW. The highest value of displacement of the fracture and von Mises stress of the internal fixation happened in 120° knee movement in both groups. The highest displacement of the DSTBW was less than that of the CSTBW (1.92 mm to 2.12 mm). The highest value of the stress on the screws was 110.60 MPa in DSTBW group, and 132.90 MPa in CSTBW group. The highest value of the stress on the titanium cable was 38.51 MPa in DSTBW group, and 41.91 MPa in CSTBW group.

**Conclusion:**

DSTBW fixation provides more stability than CSTBW fixation model in a finite element study.

## Introduction

The inferior pole patellar fractures (ITPPFs) are quite common injuries among patients suffering from multiple traumas [1]. These fractures are usually caused by a direct impact on the patella and/or a strong contraction of the quadriceps femoris muscle tissue [2]. These injuries are very painful, affect the quality of life, prolong working hours, and often lead to the loss of knee movement, which typically requires surgical treatment. Surgical options include separate vertical wiring (SVW) [3], tension band wiring [4], basket plates [5], anchor suturing [6], and cannulated screws [7].The difficulty of surgical treatment is that the displaced fracture fragments are usually small and comminuted, and it is hard to fix and maintain anatomical reduction.

**Fig. 1:**
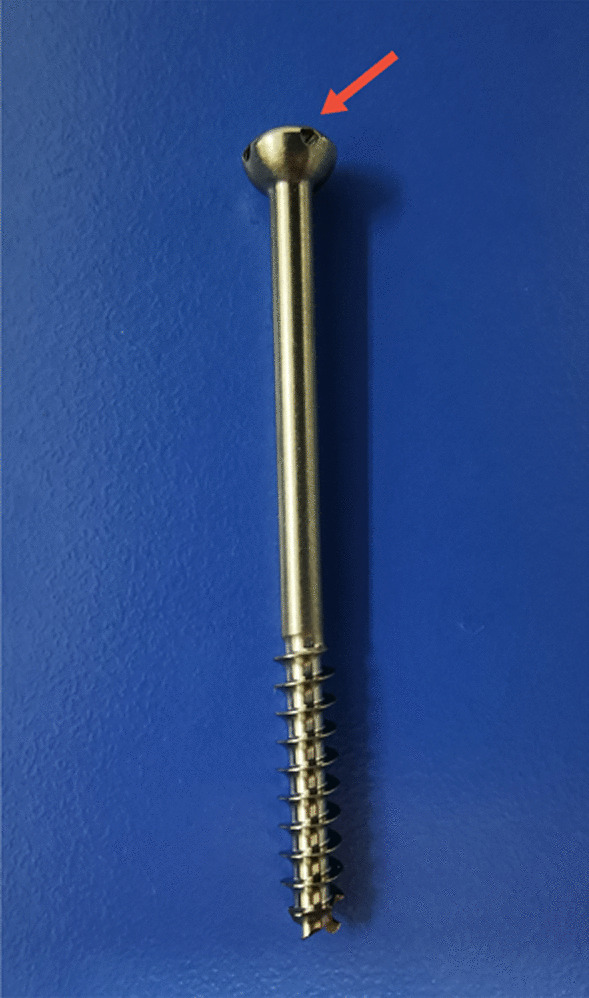
The picture of Ding’s screw. Red arrow shows the holes on the tail of Ding’s screw, allowing the ultrabraid suture to pass through

Recently, we invented a new cannulated screw with holes on the tail, which called Ding’s screw (Fig. 1). The goal of this study was to evaluate the biomechanical outcomes of this new technique (DSTBW) for the treatment of ITPPFs.

## Methods

### Finite element biomechanical study

#### Collection of imaging data

Radiologic images of a normal patella from a 27-year-old male were obtained from 0.5-mm thickness cuts of computed tomography scans to observe bone tissue and 1.0 mm thickness cuts of MRI images to observe the thickness of cartilage and ligament tissue layer.

#### Finite element model of patella construction

Radiological images were saved in DICOM format and then, imported into Mimics 17 (Materialize, Belgium) to develop a patellar 3D model. After threshold segmentation, region growth, and calculation, physical and chemical treatments were performed in software Imageware 13.0 (Siemens, Plano, TX) and Geomagic 2012 (Cary, NC) to establish a patellar model based on our previous research [8].

**Fig. 2:**
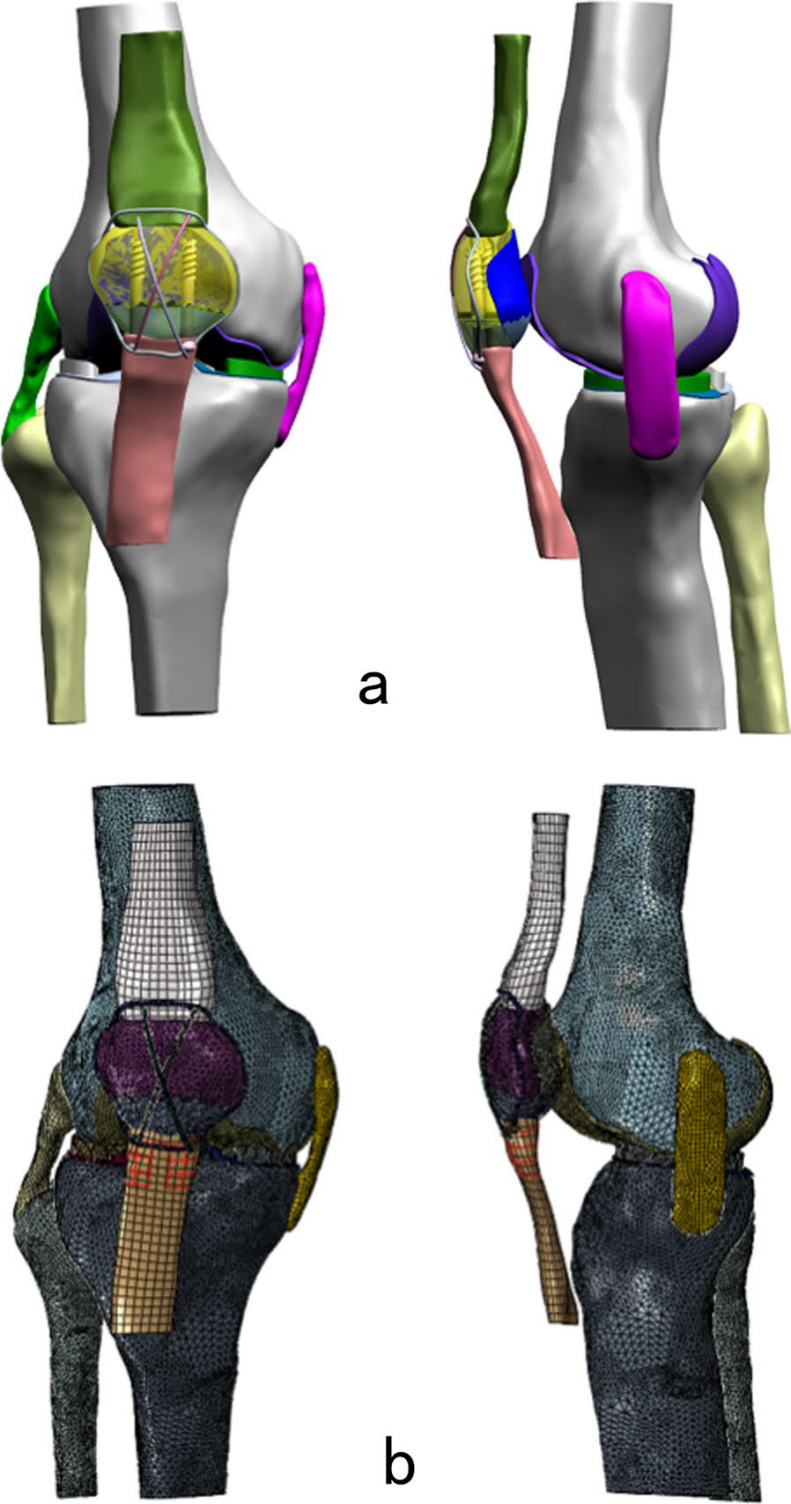
The established patellar 3D model in positive and lateral view (**a**) and the meshing of the DSTBW model in positive and lateral view (**b**)

The inferior pole patellar fracture line was created. CSTBW and DSTBW surgical procedures were applied to fix the fracture by ProE 5.0 software (PTC Inc., Boston, MA). In the CSTBW group (Fig. 2a); fractures were fixed using traditional methods, as described in previous studies [9]. In the DSTBW group (Fig. 2b), the fracture was fixed according to the method described in our previous study [9].

#### Volume mesh generation

The established model combined with the internal fixation system was output by step format and imported into the ANSYS Workbench 2020R2 software (ANSYS, Inc., Pittsburgh, PA, USA) [8]. The FE meshes were generated as a tetrahedral 1.6 mm for patella. The screws were modeled as 4 mm thick. The steel wire was modeled as 1.25 mm thick. The average mesh quality was 0.82. Overall, 152,387 nodes and 83,374 elements were used in CSTBW model; there were 146,723 nodes and 82,845 elements in DSTBW model.

#### Assignment of material properties

Material parameters (including modulus of elasticity, Poisson ratio, density of wire, and patella bone) (Table 1), contact parameters between the patella and wire, the boundary condition, and the tension load on the superior pole of the patella were defined based on previous studies [8]. The screws and wires were modeled as titanium alloy Ti–6Al–4V in this study. The average yield strength of Ti–6Al–4V is over 800 Mpa [10]. Fixation failure is defined as the highest von Mises stress exceeding the yield strength of Ti–6Al–4V.

**Table 1:** Material parameters for the bone and fixation systems

Component name	Young’s modulus (MPa)	Poisson’s ratio
Cortical bone	13,400	0.3
Cancellous bone	2000	0.3
Cartilage	20.7	0.45
Screw	210,000	0.3
Titanium cable	110,000	0.3

#### Loading and boundary conditions

An element size from 4 mm to 0.3 mm was meshed and selected a convergence analysis with 5% tolerance [8]. In the simulation, the origin of quadriceps was coupled with a concentrated force of 400 N according to the published references [11] and coincided in the direction with the muscle. The tibia and fibula were fixed, and the femur and patella moved freely. A reference loading point was set at the femoral head above the femur, and a 300 N gravity load was loaded along the force line of the knee joint to simulate the load of half of their own weight. Bend the knee joint by 30°~120°, simulating the bending motion of the knee joint.

After the mechanical loads were defined, the distribution of von Mises stress on fixation and the changes of opening and twisting angles in the fracture line were evaluated. Applying an axial displacement loading, record and draw the loading and displacement curves of K-wires or screws. The peak is considered as the maximum pullout strength.

## Results

### Finite element analysis: displacement of fractures

**Fig. 3:**
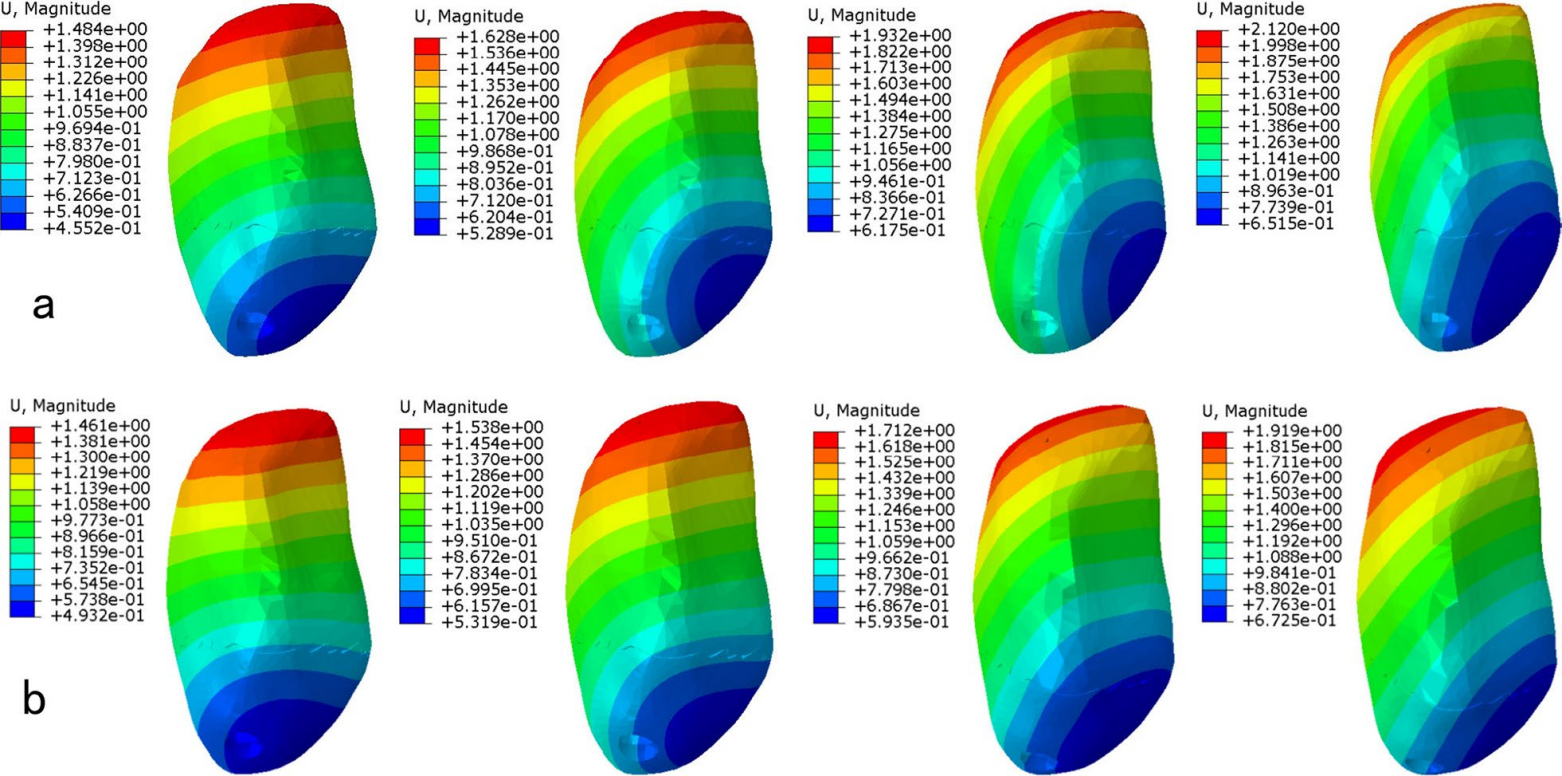
Maximum Von Mises stress of the fractured patella with CSTBW group (**a**) and DSTBW group (**b**) in different knee movement (30°, 60°, 90°, 120°) under a 400 N force

In a neutral loading of 400 N, there was less displacement of DSTBW in different knee movement (30°, 60°, 90°, 120°) when compared to CSTBW(Table 2). The highest displacement of the fractured patella happened in 120° knee movement in both CSTBW and DSTBW group, the relative displacement was as follows: 2.12 mm for the CSTBW (Fig. 3a); 1.92 mm for the DSTBW (Fig. 3b).

**Table 2:** Maximum displacement of the fractured patella with CSTBW (a) and DSTBW (b) in different knee movement under a 400 N force

Knee movement (°)	Maximum displacement of the fractured patella (mm)	
	CSTBW	DSTBW
30	1.48	1.46
60	1.63	1.54
90	1.93	1.71
120	2.12	1.92

### Finite element analysis: von Mises stress on fixation

**Fig. 4:**
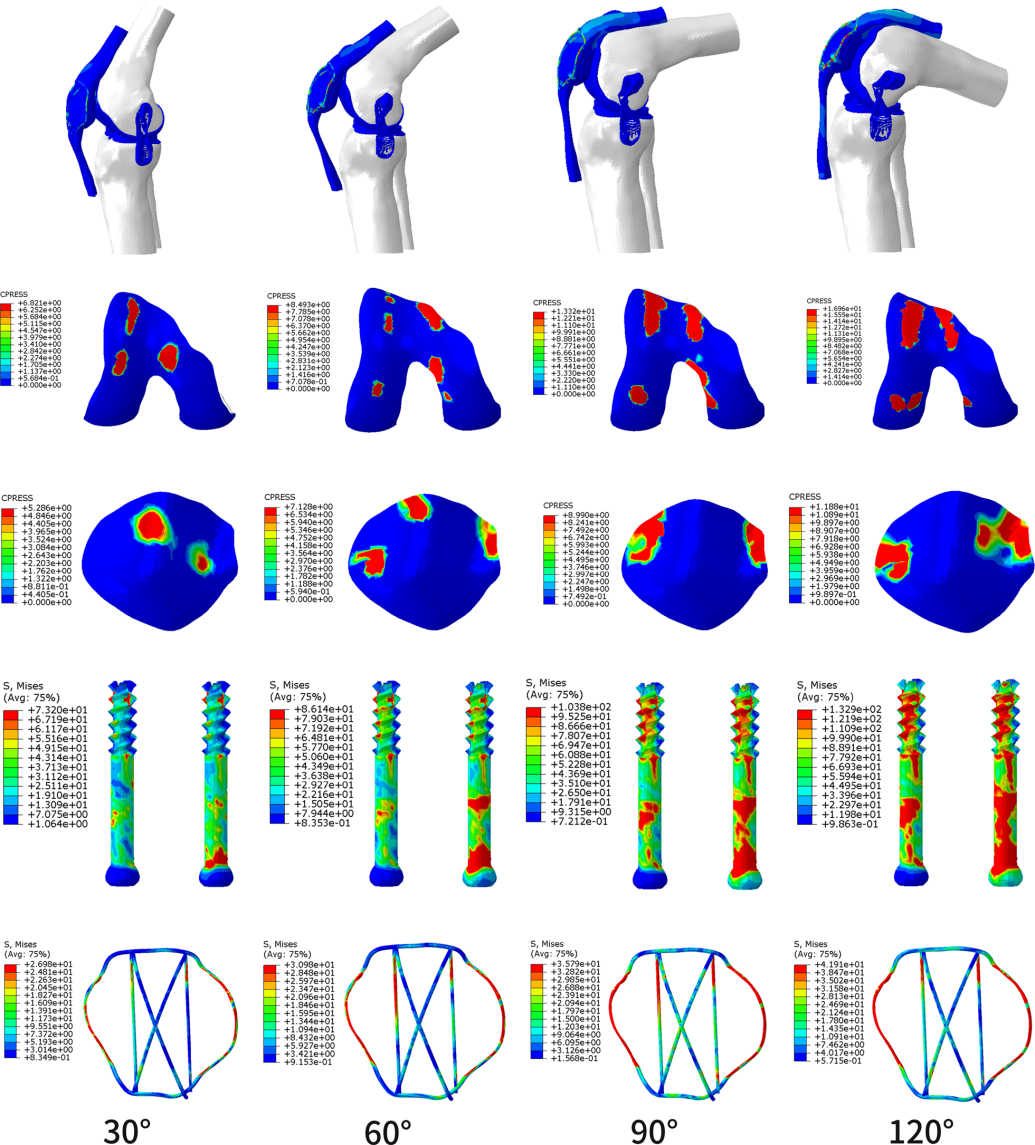
The stress distribution on the internal fixations in CSTBW group in different knee movement

**Fig. 5:**
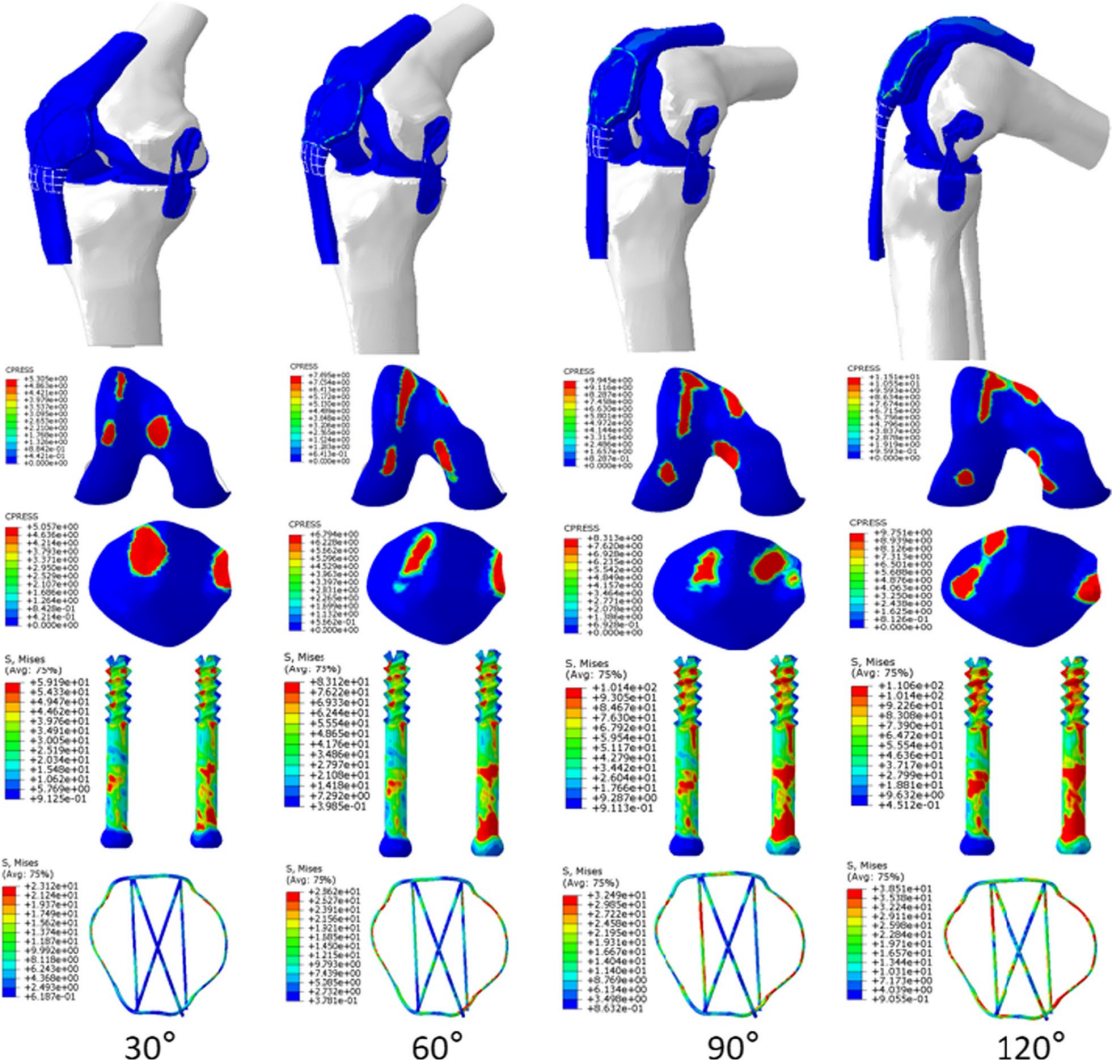
The stress distribution on the internal fixations in DSTBW group in different knee movement

There was no fixation failure in either fixation system. There was less stress distribution of DSTBW in different knee movement (30°, 60°, 90°, 120°) when compared to CSTBW (Table 3).The highest value of von Mises stress of the femur, platella, screw and titanium cable happened in 120° knee movement in both CSTBW (Fig. 4) and DSTBW group (Fig. 5). The highest value of the stress on the screws was 110.60 MPa in DSTBW group and 132.90 MPa in CSTBW group. The highest value of the stress on the titanium cable was 38.51 MPa in DSTBW group, and 41.91 MPa in CSTBW group. The highest value of the stress on the patella was 9.75 MPa in DSTBW group, and 11.88 MPa in CSTBW group.

**Table 3:** Maximum Von Mises stress (MPa) in different knee movement

Knee movement (°)	Maximum Von Mises stress (MPa)							
	CSTBW				DSTBW			
	Femur	Patella	Screw	Titanium cable	Femur	Patella	Screw	Titanium cable
30	6.82	5.29	73.2	26.98	5.31	5.06	59.19	23.12
60	8.49	7.13	86.14	30.98	7.70	6.79	83.12	28.62
90	13.3	8.99	103.80	35.79	9.95	8.31	101.40	32.49
120	16.96	11.88	132.90	41.91	11.51	9.75	110.6	38.51

## Discussion

In the present study, we performed a finite element study evaluating DSTBW and CSTBW use in the setting of ITPPFs. Our data showed the less displacement of DSTBW compared to CSTBW, which reflected significantly less gap formation with the use of DSTBW fixation with loading. The stress distribution of fracture end and internal fixation is also an important index to evaluate its effect. Ideally, the stress distribution should be evenly distributed on the internal fixation. Excessive concentration in a single area is easy to lead to the broken of the medical apparatus and instruments. In our study, the stress distribution of the femur, patella, screws and titanium cable was tested after constraint and loading on the model. The results in this finite element model showed that patella, Ding's screw and titanium cable under a less force than CSTBW in knee motion, which suggest a less chance of failure happen on the DSTBW. When these findings are applied to clinical practice, it may be postulated that DSTBW may permit earlier range of motion given the reduced gap formation and under a less force noted during loading.

The previous studies have reported tension band wiring fixation may provide stable fixation and satisfactory results [12, 13], which is considered to be the gold standard for optimal recovery of knee function [14]. When ITPPFs refer to comminuted fragments, the stability can be enhanced by adding cerclage after tension band wiring fixation. Yang et al. [12] retrospectively identified eleven patients of displaced ITPPF (AO/OTA 34-A1), treated with a modified tension band technique combined with cable cerclage, and evaluated knee function with Rasmussen scores, which achieved excellent results. The cannulated screws provide higher stability and causes less skin irritation than the conventional tension band wiring techniques using Kirschner wires [15]. Chang et al. [13] reported that satisfactory Bostman scores and Short Musculoskeletal Function Assessment dysfunction scores were obtained by using anterior tension band wiring through cannulated screws in patients of ITPPFs. However, this technique still does not provide strong fixation, especially when there are very small fragments of inferior patellar pole fractures. In previous biomechanical studies and clinical plications, bone anchor techniques have reported may provide sufficient strength for early mobilization after surgery [2, 6, 16]. Ettinger et al. [16] used 30 cadaveric specimens to study the biomechanics of ITPPFs treated with titanium/resorbable hydroxyapatite suture anchors versus transosseous tunnel repair, the results showed that the tendon repairs with suture anchors yielded significantly less gap formation during cyclic loading and resisted significantly higher ultimate failure loads compared to the transosseous tunnel repair. However, completely pullout of the eyelet within the suture anchor was a common failure. Recently, we have developed a new technology DSTBW, which like the combination of CSTBW and anchor suturing, in order to improve the effectiveness of cannulated screws and promote early exercise of knee joint.

This study has some limitations. First, it is difficult to design the holes of Ding's screw in the finite element model, so it is impossible to set the sutures and internal fixation as a whole in the DSTBW group, which may reduce the actual biomechanical effect. Second, knee is a complex joint with multiple muscles crossing over the joint and also includes synovial fluid and multiple ligamentous structures, it is hard to analysis all the soft tissue structures in a finite element model. Further biomechanical experiments include all muscle forces and the natural knee movements are needed to verify the results. This study did not investigate the distribution of Von Mises stress on the patellar tendon, which is also a limitation. The rupture of the proximal patellar ligament [17], similar to a fracture of the inferior pole of the patella, may be treated with DSTBW. Further biomechanical experiments are needed to verify this point.

## Conclusion

Our finite element analysis showed that DSTBW fixation provides more stability than the CSTBW fixation model at 30° to 120° knee flexion.

## Data Availability

All data generated or analyzed during this study are included in this published article [and its supplementary information files].
